# Glial handling of alpha-synuclein seeds: uptake, degradation and redistribution in pathological propagation

**DOI:** 10.3389/fnins.2026.2017355

**Published:** 2026-10-30

**Authors:** R. M. Meade

**Affiliations:** Department of Life Sciences, University of Bath, Bath, United Kingdom

**Keywords:** astrocytes, lysosome, microglia, neuroinflammation, Parkinson’s disease, preformed fibrils, protein propagation, α-synuclein

## Abstract

α-synuclein (αS) aggregation and propagation are central features of Parkinson’s disease pathology. Preformed fibril (PFF) models have shown that extracellular αS aggregates can enter neurons and seed aggregation of endogenous αS. Within the brain, these seeds also encounter microglia and astrocytes, which efficiently internalise fibrillar αS and can influence its subsequent fate. This review examines the mechanisms through which glial cells take up, process, degrade and redistribute extracellular αS seeds. When lysosomal trafficking and degradation are effective, glia can act as a sink for seed-competent αS and restrict neuronal seeding and propagation. In contrast, lysosomal dysfunction, incomplete proteolysis or excessive aggregate burden can promote persistence, modification and redistribution of seed-competent αS, including through extracellular vesicles and intercellular transfer. Evidence from microglial and astrocytic models therefore indicates that the outcome of glial uptake depends on the balance between internalisation, intracellular processing and subsequent release. Differences in αS conformation, fibril preparation, inflammatory state, genetic background and experimental model may further explain the apparently protective and pathogenic effects of glia reported across different studies. Understanding the factors that determine whether glial uptake results in productive clearance or continued propagation may identify therapeutic strategies that enhance the removal of pathogenic αS without broadly suppressing glial function.

## Introduction

### α-synuclein propagation is not exclusively neuronal

The accumulation of aggregated α-synuclein (αS) within Lewy bodies and Lewy neurites is a defining pathological feature of Parkinson’s disease (PD). Misfolded αS can act as a template that recruits endogenous αS leading to further aggregation. This process can be reproduced experimentally using αS preformed fibrils (PFFs), which are internalised by neurons and induce insoluble, phosphorylated αS inclusions from endogenous protein (Volpicelli-Daley et al., 2011). Injection of PFFs has also been shown to initiate progressive αS pathology in wild-type mice, including spread to anatomically connected regions and dopaminergic neurodegeneration (Luk et al., 2012). These PFF models have therefore become a key experimental system for investigating αS seeding and propagation. However, the causal relationship between αS seed-induced aggregation and neuronal degeneration remains incompletely understood, and the mechanisms underlying selective neuronal vulnerability in PD have yet to be fully resolved (Surmeier et al., 2017).

Microglia and astrocytes are major glial cell populations that help maintain the neuronal environment (Vainchtein and Molofsky, 2020). Microglia act as the resident phagocytic cells of the central nervous system, removing extracellular material and responding to cellular damage, while astrocytes support neuronal homeostasis and contribute to the uptake and processing of extracellular proteins. Both cell types are therefore positioned to encounter extracellular αS released from affected neurons before it reaches neighbouring neuronal cells.

This creates an important intermediate step in αS propagation. If an extracellular αS seed is intercepted by microglia or astrocytes and efficiently degraded, glial uptake can reduce the amount of seed-competent material available to neighbouring neurons. In contrast, if degradation is incomplete, the seed may persist, undergo further processing or be released back into the extracellular environment. Seed-competent αS can then be taken up by another neuron, where it can recruit endogenous αS and initiate further aggregation. The key issue explored here is therefore whether glia uptake ultimately terminates or redirects an αS seed.

Microglia and astrocytes both recognise and internalise aggregated αS (Lee et al., 2008; Dorion et al., 2024; Niskanen et al., 2025; Loria et al., 2017). Microglia show particularly efficient uptake and degradation of extracellular αS (Lee et al., 2008). In human primary and iPSC-derived microglia, this uptake can occur through MerTK, a receptor tyrosine kinase involved in homeostatic phagocytosis of extracellular material (Dorion et al., 2024; Niskanen et al., 2025). PFF uptake is also strongly influenced by inflammatory state (Dorion et al., 2024). Astrocytes similarly internalise fibrillar αS and traffic it to lysosomal compartments for degradation (Loria et al., 2017). These observations place glial cells at an important point between the release of an αS seed from one neuron and its potential uptake by the next.

## Glia as a sink for extracellular α-synuclein seeds

### Microglial uptake and clearance of fibrillar α-synuclein

As resident phagocytes of the central nervous system, microglia are well positioned to remove extracellular αS before it is internalised by neighbouring neurons. Early comparisons across different brain cell types have shown rapid microglial clearance of extracellular αS aggregates, supporting a scavenging role (Lee et al., 2008). More recent work has identified specific uptake pathways. In human primary and iPSC-derived microglia, MerTK mediates fibril internalisation, with inhibition or knockdown leading to reduced uptake (Dorion et al., 2024). Clearance capacity also depends on microglial state. Stimulation with the pro-inflammatory cytokine interferon-γ (IFNγ) reduces PFF uptake and disrupts phagosome maturation in human iPSC-derived microglia (Niskanen et al., 2025), indicating that inflammation may inhibit the removal of extracellular seeds.

Microglia can also cooperate to increase degradative capacity. It has been shown that αS-loaded microglia can transfer fibrillar cargo through F-actin-dependent intercellular connections to neighbouring microglia, where it is subsequently degraded. Redistribution reduced inflammatory activation and improved survival (Scheiblich et al., 2021). Evidence that microglial clearance can decrease pathology has also been observed *in vivo.* In a humanised mouse model, transplanted human iPSC-derived microglia reduced the propagation of injected PFF pathology from the striatum to the substantia nigra and preserved dopaminergic neurons (Albert et al., 2025).

### Astrocyte α-synuclein clearance

Astrocytes also internalise extracellular fibrillar αS. Neuron-to-astrocyte transfer directs fibrillar αS to lysosomal compartments, where the astrocytes degrade it more efficiently than neurons (Loria et al., 2017). Direct comparisons have shown PFF half-lives of approximately three to six hours in primary mouse microglia and astrocytes, whereas in primary mouse neurons full-length fibrils had an approximate half-life of approximately 12 h (Karim et al., 2026). *In vivo*, labelled fibrils similarly accumulated rapidly in glial cells before being cleared, compared to neurons where accumulation was slower and more persistent (Karim et al., 2026).

Astrocytic uptake does not always lead to complete clearance. In primary mouse cortical co-cultures, astrocytes internalised large amounts of oligomeric αS, but incomplete lysosomal degradation resulted in persistent intracellular deposits. The αS-loaded astrocytes also displayed mitochondrial abnormalities (Lindström et al., 2017). PD-linked genetic background can also influence astrocytic clearance, with astrocytes carrying the LRRK2 G2019S mutation showing reduced capacity to internalise and clear fibrillar αS through the endolysosomal pathway compared with wild-type astrocytes (Streubel-Gallasch et al., 2021).

This is particularly important in astrocyte-neuron co-cultures, where primary astrocytes rapidly internalise and degrade PFFs, thereby reducing aggregation of endogenous neuronal αS (Roth et al., 2026). Together, these studies support a model in which microglia and astrocytes initially act as a sink for extracellular seed-competent αS. However, this protection depends on the assumption that internalised fibrillar material is efficiently and completely degraded.

## Intracellular fate: degradation versus persistence

Following uptake, PFFs accumulate within endolysosomal compartments, where glia can rapidly degrade the fibrillar αS (Karim et al., 2026; Roth et al., 2026). However, the same material can also damage the degradative system responsible for its removal. Using murine BV2 cells and primary mouse microglia, fibrillar, but not monomeric, αS was shown to cause lysosomal damage. Autophagy inhibition under these conditions impaired mitochondrial function and reduced microglial survival (Bussi et al., 2018). More recently, PFF exposure was reported to impair microglial lysosomal acidification by binding ATP6V0C, a component of the lysosomal V-ATPase proton pump, and disrupting V-ATPase assembly. Restoring ATP6V0C was found to improve lysosomal acidification and αS degradation and reduced αS pathology in a PFF mouse model (Wang et al., 2026). This provides a direct route through which PFFs could compromise their own degradation.

### Incomplete processing may preserve or even enhance seeding activity

Delivery to the lysosome does not necessarily result in complete destruction of a seed. In a mouse PFF model, microglial TREM2-dependent uptake of αS fibrils was followed by cleavage by the lysosomal protease asparagine endopeptidase (AEP). AEP processing generated C-terminally truncated αS_1-103_ fibrils with increased seeding activity, while deletion of either TREM2 or AEP reduced pathology (Xiong et al., 2025). Partial proteolysis may therefore remodel rather than eliminate an incoming fibril, potentially increasing its seeding activity.

The importance of complete degradation is particularly clear in astrocytes. It has been found that astrocytes rapidly degraded PFFs, and reduced neuronal αS seeding, whereas pharmacological disruption of lysosomal function inhibited this protection. PFF-treated, lysosome-compromised astrocytes induced neuronal αS pathology, whereas PFF-treated astrocytes with intact lysosomes did not (Roth et al., 2026). The degradative capacity of the receiving cell therefore directly influences the subsequent fate and therefore pathology of the seed.

### Lysosomal stress can favour release rather than degradation of αS seeds

When degradation of the internalised αS seeds fails they can be redirected towards secretion. Lysosomal overload promotes release of insoluble αS from PFF-loaded macrophages and microglia through an LRRK2-Rab10-dependent pathway involving extracellular vesicles (Abe et al., 2024). It was found that PFF exposure impaired normal autophagic processing in activated microglia through PELI1-dependent degradation of LAMP2 and promoted release of αS-containing exosomes capable of seeding neuronal aggregation (Guo et al., 2020). Increased extracellular-vesicle secretion was also observed following PFF-induced impairment of lysosomal acidification (Wang et al., 2026).

These findings show that internalisation does not necessarily result in clearance. Uptake is protective only if and when the internalised seed reaches complete degradation. If the processing is incomplete or the lysosomal capacity is compromised, glial cells can retain, modify or release seed-competent αS. The glial lysosome may therefore represent a critical ‘glial checkpoint’ determining whether interception terminates or redirects propagation.

The term ‘glial checkpoint’ is used here as an operational description rather than an established model. The glial contribution becomes protective when the rate of complete degradation exceeds the rate of release of seed-competent αS aggregates, or when the released material becomes seed-incompetent. In contrast, propagation is favoured when seed-competent αS aggregates persist or are released at a rate that exceeds their complete degradation.

## Clearance or redistribution? Glial cells as intermediates in α-synuclein transfer

Failure to completely degrade internalised αS provides one route through which glia may return seed-competent material to the extracellular environment. However, αS also moves between cells through extracellular vesicles and direct intercellular connections. Transfer itself should not be considered equivalent to pathological propagation; the biological outcome depends on the recipient cell and the subsequent fate of the transferred material.

Microglia exposed to PFFs can release αS-containing exosomes that are internalised by neurons and induce aggregation of endogenous αS. Inhibition of exosome production reduced transmission, while striatal injection of exosomes from PFF-treated microglia produced phosphorylated αS pathology in anatomically connected regions (Guo et al., 2020). Together with the LRRK2-Rab10 pathway described above (Abe et al., 2024), these findings suggest that extracellular-vesicle release can return incompletely degraded αS to the surrounding neurons.

In contrast, direct intercellular transfer can actually facilitate clearance. αS-loaded microglia can redistribute fibrillar αS to neighbouring microglia, reducing the burden on individual cells and allowing the transferred material to be degraded. The same intercellular network can also transfer mitochondria towards stressed αS-loaded microglia, providing additional metabolic support (Scheiblich et al., 2021). Recent work using human neuronal and microglial models suggests that a similar process can occur between neurons and microglia. αS-induced mitochondrial damage caused release of mitochondrial DNA into the cytoplasm, activating the cGAS-STING innate immune pathway and promoting formation of tunnelling nanotube-like connections. These connections enabled directional transfer of αS aggregates and damaged mitochondria from neurons to microglia, with the transferred mitochondria subsequently targeted to microglial lysosomes for degradation (Chakraborty et al., 2026). Together, these findings suggest that direct cell-to-cell transfer can provide a route for redistributing αS and damaged cellular material towards cells with greater degradative capacity.

Direct astrocyte-to-astrocyte transfer has also been observed. Human embryonic stem cell-derived astrocytes exposed to aggregated αS transferred αS to neighbouring astrocytes through tunnelling nanotube-like connections when lysosomal degradation was insufficient. Mitochondria were transferred in the opposite direction towards stressed αS-loaded astrocytes, suggesting a compensatory response to the cellular burden (Rostami et al., 2017).

Astrocytes and microglia may also operate as a cooperative clearance network. In a co-culture of human iPSC-derived astrocytes and microglia, fibrillar αS accumulation was reduced compared with either glial cell type alone. Astrocytes accumulated more internalised αS aggregates and were more likely to subsequently release them, whereas microglia showed greater degradative capacity. Live-cell imaging also demonstrated direct transfer of αS-containing material from astrocytes to microglia (Rostami et al., 2021). These findings highlight an important distinction between αS transfer and αS propagation. Transfer to a degradation-competent glial cell can eliminate a seed, whereas release followed by neuronal uptake can return it to an environment where endogenous αS is recruited.

## Why does glial handling appear protective in some studies and pathogenic in others?

The studies described above present two sides of the same coin. Human microglia can restrict PFF propagation *in vivo* (Albert et al., 2025), while microglial depletion can also be seen to reduce propagation in other PFF models (Xiong et al., 2025; Guo et al., 2020). Astrocytes can protect neighbouring neurons through PFF clearance, yet lysosomal impairment can convert PFF-loaded astrocytes into a source of neuronal seeding (Roth et al., 2026). These differences may reflect both the type of αS assembly present and the state of the glial cell that encounters it.

### Not all α-synuclein assemblies are equivalent

Aggregated αS is structurally heterogeneous. Previous work has compared oligomeric species, PFFs and mature fibrils in human iPSC-derived microglia and found distinct transcriptional and inflammatory responses. Mature fibrils produced particularly strong inflammatory activation, while conventional PFFs caused comparatively limited acute cytokine induction. Sonication also showed altered PFF structure and TLR4 activation (Chang et al., 2025). Disease-derived assemblies may differ further from recombinant PFF preparations. Fibrillar αS amplified from PD brain induced stronger cytokine and glutamate responses in primary microglia than recombinant fibrils (Yildirim-Balatan et al., 2024). “Aggregated αS” should therefore not be treated as a single biological stimulus.

### PFF preparation can substantially alter biological activity

Variation exists even between preparations described as the same PFF species. Increasing sonication initially enhances seeding as fibrils fragment, whereas prolonged sonication disrupts fibrillar β-sheet structure and reduces seeding (Ohira et al., 2025). Guidance on preparation of PFFs *in vitro* emphasises control of monomer preparation, aggregation conditions, storage, sonication, fibril size and biochemical validation (Polinski et al., 2018; MJFF, 2017). Concentration and duration of exposure provide additional variables. A fibrillar burden that is rapidly cleared is unlikely to have the same consequence as prolonged delivery to the lysosomal compartment. “PFF exposure” therefore describes an experimental category rather than a standardised condition, and comparisons between studies should account for the preparation used.

### The state and origin of the glial cell are equally important

The receiving cell is not static, and its functional state can vary widely. In human microglia, activation with the pro-inflammatory cytokine interferon-γ (IFNγ) reduces PFF uptake and disrupts phagosome maturation (Niskanen et al., 2025), whereas MerTK-mediated uptake can occur without a strong pro-inflammatory response (Dorion et al., 2024). Genetic background can further shift this balance. The PD-associated LRRK2 G2019S mutation impairs the ability of microglia to redistribute fibrillar αS between neighbouring cells through intercellular connections, reducing this cooperative clearance mechanism (Scheiblich et al., 2021), while TREM2-dependent uptake can route fibrils towards AEP-dependent processing and increased seeding activity (Xiong et al., 2025).

Microglia and astrocytes should also not be considered as fixed phenotypes. Microglia occupy a range of transcriptional and functional states shaped by age, local tissue signals, inflammation and disease, while reactive astrocytes similarly show diverse molecular and functional responses rather than simple protective or pathogenic states (Paolicelli et al., 2022; Escartin et al., 2021). This is relevant to αS handling because changes in phagocytosis, lysosomal function, cytokine release and intercellular communication may alter the fate of an internalised seed.

PD genetics provides additional support for a role of glial and lysosomal pathways. Common-variant PD heritability is enriched in microglial open chromatin, with P2RY12 nominated as a candidate microglial risk locus (Andersen et al., 2021). In parallel, GBA1 variants, a major genetic risk factor for PD, reduce lysosomal glucocerebrosidase activity; reduced GCase activity in neurons and glia has been linked to αS accumulation and intercellular spread (Gegg et al., 2022). Together with LRRK2, these findings connect PD genetic risk to pathways that may alter how glial cells process αS.

Model choice may also contribute to the different outcomes reported. Transplanted human microglia have been shown to restrict PFF propagation *in vivo* (Albert et al., 2025). By contrast, microglial depletion or inhibition reduced αS propagation in mouse PFF models (Guo et al., 2020; Lai et al., 2024), while disruption of microglial TREM2-dependent processing also reduced pathology in a separate PFF model (Xiong et al., 2025). However, prolonged inhibition of colony-stimulating factor 1 receptor (CSF1R), which is required for microglial survival, depleted microglia in a rat PFF model without altering αS inclusion burden or nigrostriatal degeneration (Stoll et al., 2024). These findings are not necessarily contradictory. Instead, the effect of glia is likely to depend on the balance between αS uptake, intracellular processing and subsequent release, with the outcome influenced by both the properties of the seed and the state of the receiving cell.

## Discussion and future directions: glia as a checkpoint in α-synuclein propagation

Taken together, the evidence supports viewing glial handling as a functional ‘checkpoint’ in αS propagation, with the outcome determined by the balance between complete degradation and persistence or release of seed-competent material. When degradative capacity is sufficient, uptake by microglia and astrocytes reduces extracellular seed-competent αS and limits neuronal exposure (Albert et al., 2025; Karim et al., 2026; Roth et al., 2026). Microglial selective autophagy similarly promotes degradation of neuron-released αS, with disruption increasing αS accumulation and dopaminergic neurodegeneration (Choi et al., 2020). However, when lysosomal capacity is exceeded or compromised, internalised αS can persist, undergo pathogenic processing or be released (Roth et al., 2026; Wang et al., 2026; Xiong et al., 2025; Abe et al., 2024; Guo et al., 2020). The distinction is therefore not simply uptake versus release, but productive clearance versus non-productive processing (see Figure 1).

**Figure 1:**
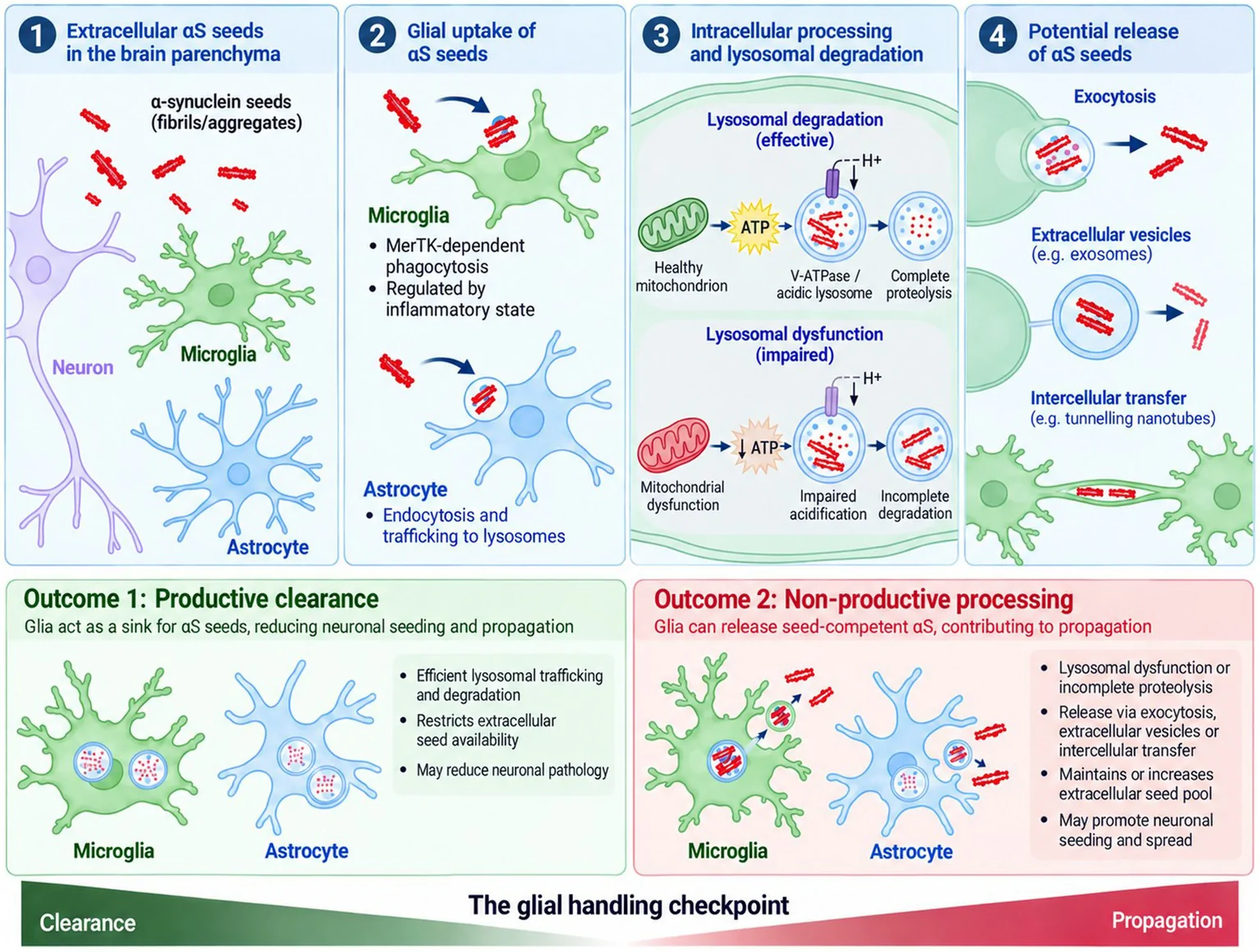
Glial handling of extracellular α-synuclein seeds as a determinant of pathological propagation. Extracellular α-synuclein (αS) seeds released from neurons can be internalised by microglia and astrocytes and subsequently trafficked through endolysosomal pathways. When lysosomal function is maintained, internalised αS can be efficiently degraded, reducing the extracellular pool of seed-competent material and limiting further neuronal seeding. In contrast, impaired lysosomal acidification, incomplete proteolysis or insufficient degradative capacity can allow αS to persist and favour its redistribution through extracellular vesicles or direct intercellular transfer. These alternative outcomes define a glial handling checkpoint in which productive clearance limits propagation, whereas non-productive processing can return seed-competent αS to the extracellular environment and promote further spread of pathology.

An important distinction should be made between αS seeding, the accumulation of pathological aggregates and neurodegeneration. PFF models show that seeded αS aggregation can be associated with neuronal dysfunction and dopaminergic neuronal loss, but the mechanisms linking these processes remain incompletely understood. The extent of αS pathology also does not necessarily reflect the severity of neurodegeneration (Surmeier et al., 2017). Neuronal vulnerability is likely to depend on additional factors, including mitochondrial dysfunction (Bussi et al., 2018; Chakraborty et al., 2026), impaired lysosomal and autophagic proteostasis (Bussi et al., 2018; Wang et al., 2026), inflammatory responses (Niskanen et al., 2025; Yildirim-Balatan et al., 2024), changes in neuron–glia interactions (Scheiblich et al., 2021; Chakraborty et al., 2026; Rostami et al., 2017) and, particularly in dopaminergic neurons, cytosolic dopamine oxidation and the generation of reactive dopamine metabolites (Masato et al., 2019; Burbulla et al., 2017). The ability of glial cells to restrict αS propagation should therefore not automatically be considered equivalent to protection from neurodegeneration.

### Metabolic coupling and glial degradative capacity

Cellular metabolic state may provide an additional link between αS burden and the ability of glia to clear an internalised seed. αS accumulation has been associated with mitochondrial damage in astrocytes and in human neuron–microglia models (Lindström et al., 2017; Chakraborty et al., 2026; Rostami et al., 2017), while fibrillar αS can damage microglial lysosomes and failure to resolve this damage impairs mitochondrial function (Bussi et al., 2018). Lysosomal acidification is maintained by the ATP-dependent V-ATPase and may therefore be sensitive to reduced cellular energy availability. Consistent with this, PFF exposure can disrupt V-ATPase assembly and impair lysosomal acidification (Wang et al., 2026). In contrast, stressed αS-loaded microglia and astrocytes can receive mitochondria from neighbouring cells through intercellular connections (Scheiblich et al., 2021; Rostami et al., 2017), suggesting that metabolic support between glial cells may help maintain their degradative capacity. Together, these observations suggest a potentially self-reinforcing relationship in which αS burden, mitochondrial dysfunction and impaired lysosomal acidification reduce complete degradation and favour persistence or release of seed-competent material.

### Therapeutic implications

This distinction has important therapeutic consequences. Broad suppression or depletion of microglia may reduce inflammatory or propagation-promoting pathways under some conditions, but could simultaneously remove an important mechanism for extracellular αS clearance. On the other hand, increasing glial uptake alone may be ineffective or detrimental if downstream degradation is already limiting.

A more selective strategy would increase the probability that internalised αS reaches a terminal degradative fate. Restoring ATP6V0C, a component of the lysosomal V-ATPase proton pump, or activating transcription factor EB (TFEB), a major regulator of lysosomal and autophagic function, improved microglial lysosomal acidification and αS degradation and reduced pathology in a PFF model (Wang et al., 2026). Similarly, intact astrocytic lysosomal function determines whether PFF uptake suppresses or promotes neuronal seeding (Roth et al., 2026). Enhancing lysosomal acidification, autophagic processing or lysosomal resilience may therefore provide a more precise approach than broadly altering glial activation.

Several questions remain unresolved. It is not known what proportion of glial-internalised αS is completely destroyed *in vivo*, how often partially degraded fibrils retain or acquire seeding activity, or how this balance changes with ageing and disease progression. It will also be important to determine whether distinct αS strains differ in susceptibility to glial degradation and how closely PFF models reproduce the handling of disease-derived αS. These questions require experiments that follow both the fate and seeding competence of internalised αS over time rather than measuring uptake or inflammatory activation alone.

The progression of αS pathology may therefore depend not only on movement of seeds between neurons, but also on whether surrounding glial cells intercept and permanently eliminate them. Defining the factors that determine the fate of an αS seed following glial uptake may reveal a tractable point of intervention between release from one neuron and recruitment of αS in the next.
